# Application of Gamma Ray-Responsive Genes for Transcriptome-Based Phytodosimetry in Rice

**DOI:** 10.3390/plants10050968

**Published:** 2021-05-13

**Authors:** Jin-Hong Kim, Kwon Hwangbo, Eujin Lee, Shubham Kumar Dubey, Moon-Soo Chung, Byung-Yeoup Chung, Sungbeom Lee

**Affiliations:** 1 Advanced Radiation Technology Institute, Korea Atomic Energy Research Institute, 29 Geumgu-gil, Jeongeup-si 56212, Korea; kwonhb@kaeri.re.kr (K.H.); dldbwls10@daum.net (E.L.); shubham26@kaeri.re.kr (S.K.D.); mschung@kaeri.re.kr (M.-S.C.); bychung@kaeri.re.kr (B.-Y.C.); sungbeom@kaeri.re.kr (S.L.); 2 Department of Radiation Science and Technology, University of Science and Technology, 217 Gajeong-ro, Yuseong-gu, Daejeon 34113, Korea

**Keywords:** gamma radiation, phytodosimetry, rice, *Arabidopsis*, transcriptome, genotoxicity

## Abstract

Transcriptome-based dose–response curves were recently applied to the phytodosimetry of gamma radiation in a dicot plant, *Arabidopsis thaliana*, as an alternative biological assessment of genotoxicity using DNA damage response (DDR) genes. In the present study, we characterized gamma ray-responsive marker genes for transcriptome-based phytodosimetry in a monocot plant, rice (*Oryza sativa* L.), and compared different phytodosimetry models between rice and *Arabidopsis* using gamma-H2AX, comet, and quantitative transcriptomic assays. The transcriptome-based dose–response curves of four marker genes (*OsGRG, OsMutS*, *OsRAD51*, and *OsRPA1*) were reliably fitted to quadratic or exponential decay equations (*r*^2^ > 0.99). However, the single or integrated dose–response curves of these genes were distinctive from the conventional models obtained by the gamma-H2AX or comet assays. In comparison, rice displayed a higher dose-dependency in the comet signal and *OsRAD51* transcription, while the gamma-H2AX induction was more dose-dependent in *Arabidopsis*. The dose-dependent transcriptions of the selected gamma-ray-inducible marker genes, including *OsGRG*, *OsMutS*, *OsRAD51*, and *OsRPA1* in rice and *AtGRG*, *AtPARP1*, *AtRAD51*, and *AtRPA1E* in *Arabidopsis*, were maintained similarly at different vegetative stages. These results suggested that the transcriptome-based phytodosimetry model should be further corrected with conventional genotoxicity- or DDR-based models despite the high reliability or dose-dependency of the model. In addition, the relative weighting of each gene in the integrated transcriptome-based dose–response model using multiple genes needs to be considered based on the trend and amplitude of the transcriptional change.

## 1. Introduction

The nuclear accidents at Chernobyl and Fukushima have greatly deepened public concern about environmental contamination from artificial radionuclides in many countries, especially in those operating nuclear power plants. Environmental radioactive substances can be transferred into the human body through non-human biota that constitute the food chain in the ecosystem [1]. Therefore, it is necessary to develop a dedicated biological risk assessment and dosimetry model based on the relationship between radiation dose and its effects using a variety of reference animals and plants [2,3]. In this regard, plants are considered to be more suitable reference biota for environmental risk assessment due to their immobility compared with animals as well as the accumulated data regarding the biological effects of ionizing radiation in various plant species [4].

Conventional biological dosimetry (biodosimetry) of ionizing radiation has utilized the gold-standard methods of dicentric chromosome and micronucleus assays to establish a dose–response curve based on chromosomal aberration since the 1960s [5,6,7]. In addition, γH2AX and single-cell gel electrophoresis (comet) assays have been adopted for biodosimetry to quantify the DNA damage response (DDR) associated with chromosomal aberration [8,9,10,11]. However, these conventional methods need to be complemented with more economic and efficient methods for plant biodosimetry (phytodosimetry) [4].

Radiation-specific transcriptomes are some of the most promising potential biomarkers that highlight the detrimental biological effects of ionizing radiation [12,13]. The degree of DNA damage can be quickly and accurately quantified by quantitative real time polymerase chain reaction (RT-qPCR) analysis [14]. Our recent study showed that an alternative transcriptome-based dose–response model for *Arabidopsis thaliana*, a dicot model plant, could be practically applied to phytodosimetry, the biological dosimetry of ionizing radiation in plants [4]. However, since there is a large difference between the genome in monocot and dicot plants [15,16,17], the availability of such transcriptome-based phytodosimetry needs to be further substantiated in monocot plants. Although DDR genes common to eukaryotic cells are preferentially considered for phytodosimetry, the radiation-responsive DDR genes of *Arabidopsis* may be not effective in rice (*Oryza sativa*), a monocot model plant. In addition, the coverage of phytodosimetry for environmental risk assessment of radioactive substances in the ecosystem will be expanded greatly by adding monocot plants. Despite a number of genetic studies in rice, until recently, radiation-responsive transcriptomic profiles had never been explored at the whole genome level after in planta exposure of monocot plants to ionizing radiation.

A recent report revealed the genome-wide radiation-responsive transcriptomic profiles in rice via RNA-seq analysis after gamma irradiation and suggested that the radiation-responsive transcriptomes in rice need to be functionally verified before being used for phytodosimetry due to the substantial difference in the expression of the DDR genes compared to those in *Arabidopsis* [18]. In this study, we selected several radiation-responsive marker genes, including DDR genes based on the rice RNA-seq data, and analyzed the dose–response curves of their transcriptomes for phytodosimetry in rice after gamma irradiation below tens of Gy. The transcriptomic changes in the marker genes after gamma irradiation were compared with the dose-dependent induction of DNA damages revealed by γH2AX and comet assays as dose–response models. The importance of using plant species-specific genes as well as those common to both monocot and dicot plants was discussed in terms of transcriptome-based phytodosimetry for the environmental risk assessment of ionizing radiation.

## 2. Results and Discussion

### 2.1. Dose–Response Curves of the DNA Damage Response (DDR) in Rice after Gamma Irradiation

**Figure 1:**
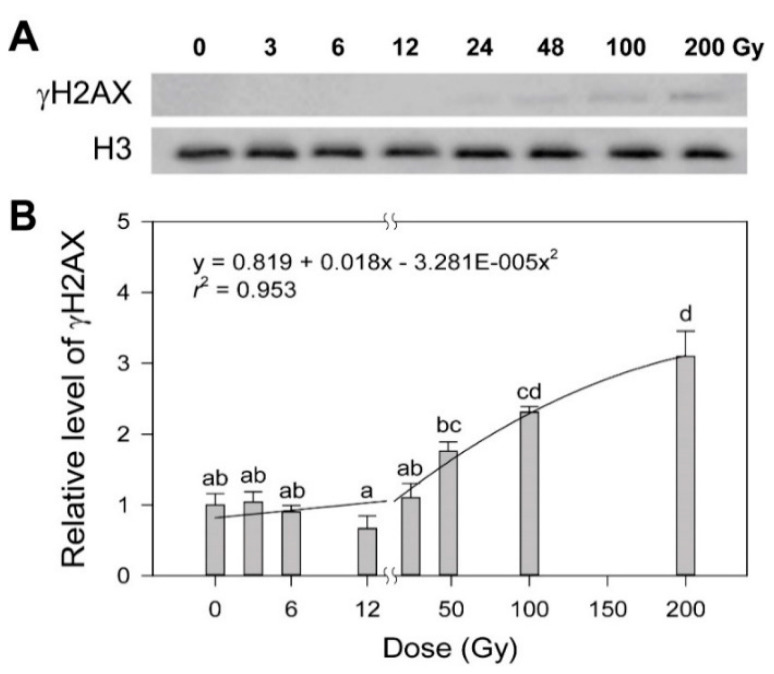
Induction of the γH2AX protein in rice irradiated with different doses of gamma radiation. The representative images (**A**) were obtained by Western blot analysis and subjected to regression for the quadratic dose–response curve (**B**). H3 was used as a loading control. Data are reported as the mean ± standard error (SE) of three independent experiments. Different letters indicate significant differences at *p* < 0.05 (one-way analysis of variance followed by Tukey’s honestly significant difference test).

Ionizing radiation, a potent genotoxic agent, causes physiological alterations as well as DNA damages including strand breaks, base modification and crosslinking [19,20,21]. Some photosynthesis-associated chlorophyll fluorescence parameters such as non-photochemical quenching and the photosynthesis performance index are known to be substantially decreased by gamma radiation below tens of Gy [22,23]. However, since there is no dose–response model for such a dose range available based on plant physiology that includes photosynthesis, we performed γH2AX and comet assays to estimate the dose-dependency of DDR in rice upon exposure to various doses of gamma radiation from 3 to 200 Gy, as previously reported in *Arabidopsis* [4]. When histone H2AX phosphorylation (γH2AX), an initial response to induced DNA double strand breaks (DSBs), was evaluated by immunoblotting, the γH2AX protein was undetectable below 24 Gy, but increased dose-dependently from 24 to 200 Gy (Figure 1A). The dose–response of γH2AX was fitted to the linear-quadratic model:

*y* = c + β*x* + α*x*^2^ (1)

where *y* is the response, *x* is the radiation dose, c is the background induction level, β is the linear component of the curve, and α is the quadratic portion of the curve. As a result, the dose–response equation of γH2AX induction as follows (Figure 1B):

*y* = 0.819 + 0.018*x* − 0.00003281*x*^2^ (*r*^2^ = 0.953) (2)

Although this equation represented a conventional dose–response curve for DDR in rice, it demonstrated relatively low confidence due to the lack of detectable γH2AX below 24 Gy, compared to that with *r*^2^ = 0.99 in *Arabidopsis* [4].

**Figure 2:**
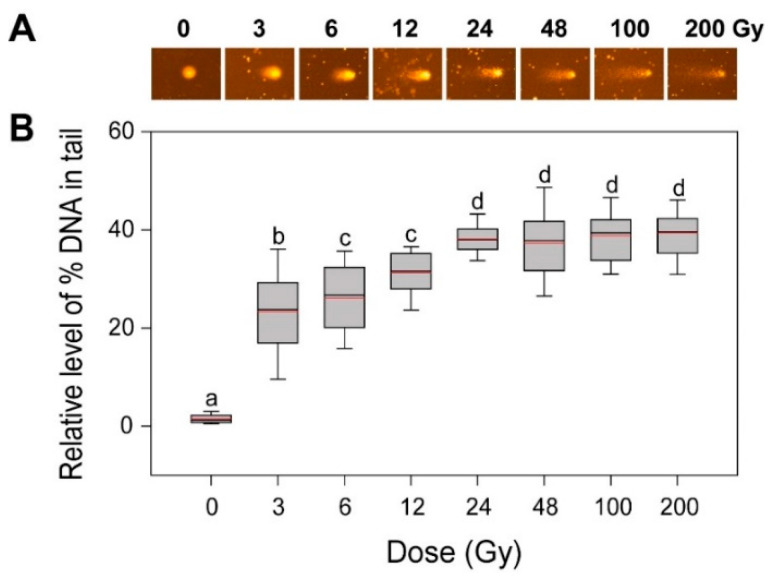
DNA damage in rice irradiated with different doses of gamma radiation. The representative images (**A**) were obtained by an alkaline comet assay and subjected to calculation for the ‘% DNA in tail’ (**B**) by Komet 5.5 image analysis software (Kinetic Imaging, Ltd., Liverpool, UK). The box plots extend from the 25th to the 75th percentiles, with black and red horizontal lines at the median (50th percentile) and the mean values, respectively. Error bars indicate the 90th and 10th percentiles. Data show the distribution of 80~160 nuclei from three independent experiments. Different letters indicate significant differences at *p* < 0.05 (one-way analysis of variance followed by Tukey’s honestly significant difference test).

Since DSBs are the most deleterious type of DNA damage but are rapidly recoverable by homologous recombination (HR) or non-homologous end-joining, they can be substantially detectable only within 1 h after irradiation [24,25,26]. The alkaline comet assay method measures various DNA damages including frank strand breaks, incomplete excision repair sites, alkali-labile sites, and crosslinking, while the neutral method mainly detects DSBs [8,26,27]. Therefore, we performed an alkaline comet assay to investigate the dose–response of the gamma-ray-induced DNA damages in rice. The ‘% DNA in tail’ increased somewhat dose-dependently up to 24 Gy, but it remained constant in the range of 24 to 200 Gy (Figure 2). When compared with the more limited dose-dependency of the ‘% DNA in tail’ below 6 Gy in *Arabidopsis* [4], the dose–response of the ‘% DNA in tail’ in rice appeared greater than that of γH2AX below 24 Gy. However, the relatively low confidence of the γH2AX assay with no γH2AX signal below 24 Gy and the saturated ‘% DNA in tail’ signal above 24 Gy in the alkaline comet assay demonstrated that DNA damage-based conventional methods such as γH2AX and comet assays may be not appropriate for phytodosimetry in rice. Moreover, the conventional models for phytodosimetry of gamma radiation showed a substantial difference in the DDR between the monocot rice and the dicot *Arabidopsis*. The difference in the DDR between rice and *Arabidopsis* may be associated with the differential expression of some DDR genes such as *RPA1*, *BRCA1*, and *CYCB1;1* after gamma irradiation as previously reported [18].

### 2.2. Transcriptional Changes of Rice Gamma Ray-Responsive Genes According to the Radiation Dose

**Figure 3:**
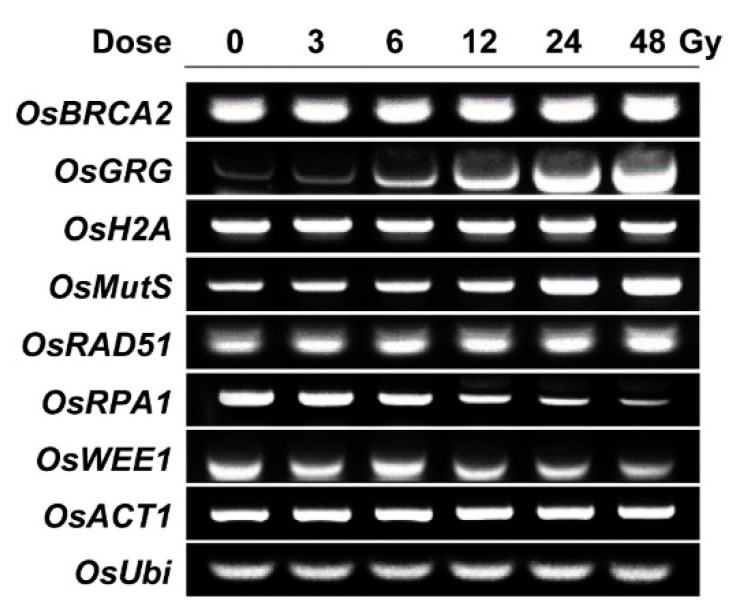
Transcription levels of gamma-ray-responsive genes in rice irradiated with different doses of gamma radiation. *OsBRCA2*, *OsGRG*, *OsH2A*, *OsMutS*, *OsRAD51*, *OsRPA1*, and *OsWEE1* were selected as gamma ray-responsive genes from our previous study [18]. Seedlings were irradiated with gamma radiation for 1 h at a dose rate of 3, 6, 12, 24, or 48 Gy h^−1^, and then they were subjected to the reverse transcription polymerase chain reaction (PCR) assay as described in the Materials and Method section. *OsACT1* and *OsUbi* were used as endogenous control genes.

To acquire a transcriptome-based dose–response or phytodosimetry model of gamma radiation in rice, we selected seven gamma-ray-responsive genes from the genome-wide rice transcriptome profile obtained after gamma irradiation of 300 Gy at a dose rate of 60 Gy h^−1^ for 5 h [18]. The seven selected genes included *OsBRCA2*, *OsGRG, OsH2A*, *OsMutS*, *OsRAD51*, *OsRPA1*, and *OsWEE1*, which showed relatively high transcriptional fold changes of −3.64, 11.09, −15.36, 3.98, 12.92, −9.40, and −2.94, respectively. *OsRAD51*, *OsRPA1*, and *OsBRCA2* are expected to participate in HR repair, *OsMutS* in mismatch repair, and *OsWEE1* in cell cycle arrest [28,29,30,31,32,33]. These were selected as DDR genes. In contrast, *OsH2A* encodes the histone H2A protein, and *OsGRG* is associated with a hypothetical protein. As previously shown by *AtGRG* [4], *OsH2A* and *OsGRG* were chosen for a reliable dose–response model due to their high and sustainable transcriptional changes after gamma irradiation. In the present study, the applicability and reliability of the seven gamma-ray-responsive genes were further evaluated for phytodosimetry after gamma irradiation at relatively low doses of 3 to 48 Gy using RT-PCR analysis. Gamma irradiation with 3, 6, 12, 24, or 48 Gy for 1 h induced dose-dependent transcriptional increases in *OsRAD51*, *OsGRG*, and *OsMutS* or decreases in *OsRPA1* and *OsWEE1*, while the expressions of *OsBRCA2* and *OsH2A* were rarely changed by the low doses of gamma radiation (Figure 3). These results demonstrated that the trends or intensities of transcriptional changes after gamma irradiation were different between the doses of 300 and 3~48 Gy. *OsRAD51*, *OsGRG*, *OsMutS*, and *OsRPA1* may be more reliable gamma-ray-responsive genes in the dose range of 3~48 Gy than *OsBRCA2* and *OsH2A*. In addition, the transcriptional changes of *OsBRCA2* and *OsRPA1* in Figure 3 were discriminated from those of *AtBRCA1* and *AtRPA1E* in our previous study [4]. The seemingly substantial difference in the transcriptional changes in the selected DDR genes between rice and *Arabidopsis*, probably due to their varied radiation sensitivities, makes it difficult to develop an integrated phytodosimetry model based on DDR marker genes common to both plant species. In contrast, gamma-ray-responsive and species-specific genes such as *OsGRG* and *AtGRG* encoding a different hypothetical protein may be useful in improving transcriptome-based phytodosimetry models in plants by correcting the differential transcriptional changes in DDR genes common to both monocot and dicot plants.

### 2.3. Dose–Response Curves of the Selected Gamma Ray-Responsive Genes in Rice after Gamma Irradiation

**Figure 4:**
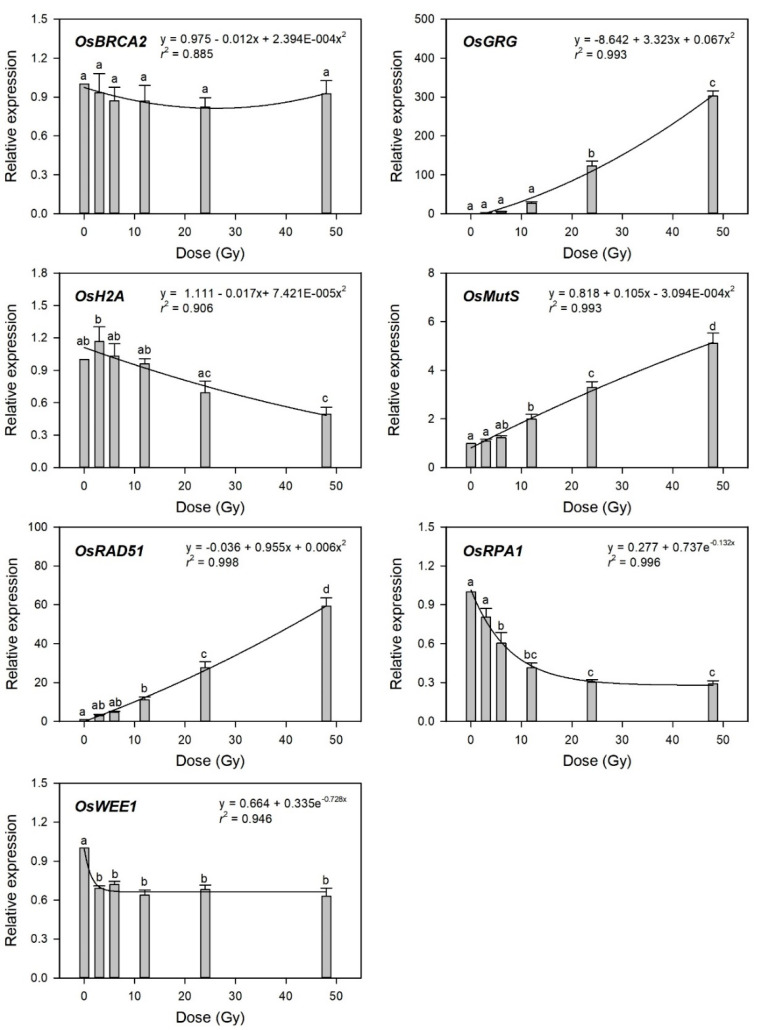
Relative transcription levels of the selected gamma-ray-responsive genes and dose–response curves after irradiation as measured by quantitative real-time polymerase chain reaction (RT-qPCR). All transcription levels are shown relative to the 0 Gy group of Dongjin-byeo seedlings. Data are the mean ± SE with *n* = 9 from three independent experiments. Different letters indicate significant differences at *p* < 0.05 (one-way analysis of variance followed by Tukey’s honestly significant difference test).

To evaluate transcriptome-based phytodosimetry models in rice, we generated quantitative dose–response curves of the selected gamma-ray-responsive genes and fitted them to different nonlinear regression equations. Gamma-ray-induced transcription of DDR genes was recently shown to depend on both the radiation dose and dose rate [4]. In the present study, various irradiation doses reflected different dose rates in 1 h of irradiation time. Since the radiation dose is proportional to the dose rate in a fixed irradiation time, it is unlikely to distinguish the former effects from the latter ones in biodosimetry or phytodosimetry. As expected by Figure 3, the gamma-ray-responsive transcriptional changes of *OsBRCA2*, *OsH2A*, and *OsWEE1* were fitted to quadratic or exponential decay equations with a low coefficient of determination (*r*^2^ < 0.95), while those of *OsGRG, OsMutS*, *OsRAD51*, and *OsRPA1* represented more reliable dose-dependent equations with *r*^2^ > 0.99 (Figure 4).

**Figure 5:**
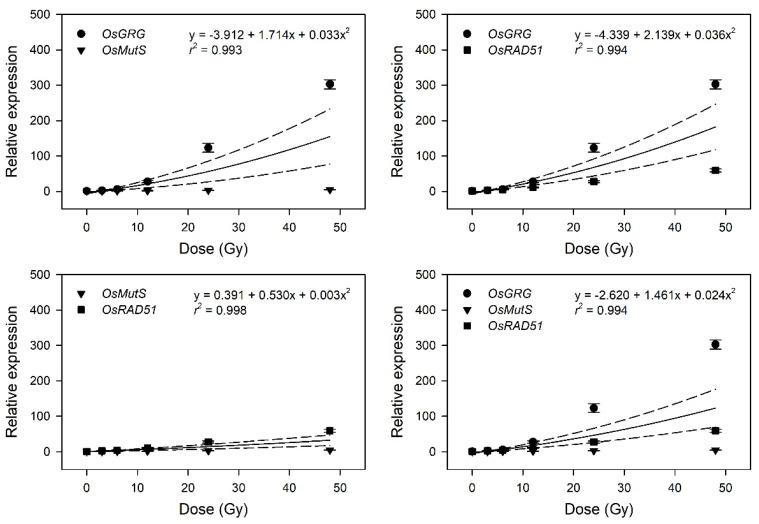
Dose–response curves pooled from the relative transcription levels of three selected gamma-ray-responsive genes. The dose–response curves of *OsGRG*, *OsMutS*, and *OsRAD51* were integrated as a linear-quadratic equation. The solid line is the linear regression fitted line with *r*^2^ values, and the dashed lines show the upper and lower 95% confidence intervals.

This result implies that the latter four genes may be considered as marker genes for transcriptome-based phytodosimetry of gamma radiation in rice. However, these genes displayed a lack of reliable statistical difference or dose-dependency of transcriptional changes in the dose range of 3–12 Gy. Therefore, two or three dose–response curves of *OsGRG, OsMutS*, and *OsRAD51* with a linear or supra-linear quadratic equation were integrated to compare the reliability or statistical confidence of the transcriptome-based dose–response models based on multiple genes (Figure 5). The integrated dose–response models displayed a reliable quadratic equation (*r*^2^ > 0.99) but demonstrated an increased dependency for the genes (*OsGRG* > *OsRAD51* > *OsMutS*) with higher transcriptional induction. This indicates that the relative weighting or contribution of each gene in the integrated transcriptome-based dose–response model using multiple genes needs to be corrected based on the trend and amplitude of transcriptional change. In addition, the species-specific marker genes displaying distinctive transcriptional changes between rice and *Arabidopsis* may contribute to reliability and differentiation of transcriptome-based models in monocot and dicot plants.

### 2.4. Distinctive Genotoxicity- and Transcriptome-Based Dose–Response Curves between Rice and Arabidopsis

**Figure 6:**
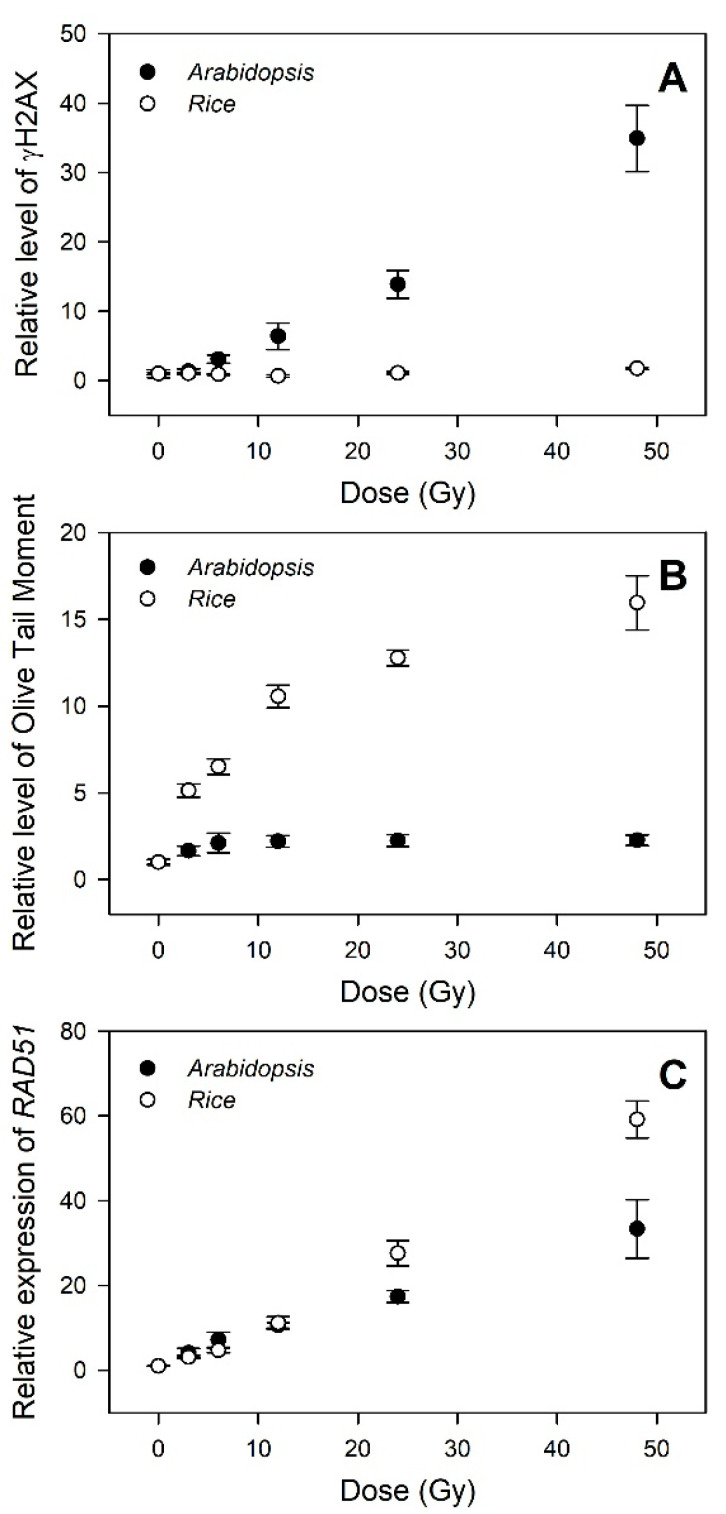
Difference in the DNA damage response (DDR)-based conventional and transcriptional dose–response curves between rice and *Arabidopsis*. (**A**–**C**) represent the relative levels of γH2AX, olive tail moment (OTM), and *RAD51* transcription, respectively. All data are shown relative to the 0 Gy group. The data for *Arabidopsis* were obtained from our previous study [4]. The OTM values were calculated as (Tail.mean – Head.mean) × Tail % DNA/100 by Komet 5.5 image analysis software (Kinetic Imaging, Ltd., Liverpool, UK).

The genotoxicity- and transcriptome-based dose–response curves generated by the γH2AX, comet, and RT-qPCR assays were further compared to evaluate the relative confidence of different phytodosimetry models in rice and *Arabidopsis*. The dose–response curves of γH2AX induction displayed a high dose-dependency in *Arabidopsis* within the dose range of 3–48 Gy, but almost no change in rice (Figure 6A). In contrast, the olive tail moment (OTM), which was shown as the most informative parameter of the comet image for radiation-induced DNA damage in mammalian cells [34], represented a much more dose-dependent hyperbolic dose–response curve in rice compared to that in *Arabidopsis* (Figure 6B). This result may suggest that major types or intensities of DNA damages caused by gamma irradiation are different between rice and *Arabidopsis*. Compared with the two genotoxicity-based curves, the transcriptome-based dose–response curves using *RAD51*, a representative DDR gene, commonly displayed high dose-dependency in both rice and *Arabidopsis*, with a greater dose–response in the former (Figure 6C). Therefore, the transcriptome-based dose–response models using the general DDR marker genes are unlikely to be strongly correlated with an actual dose-dependency of genotoxicity or DNA damage. Although these models would be more applicable for phytodosimetry due to the more reliable and higher dose-dependency, they need to be more consistent with the genotoxicity-based conventional dose–response models.

### 2.5. Transcriptional Variation of the Selected Gamma-Ray-Responsive Genes in Rice and Arabidopsis at Different Developmental Stages

**Figure 7:**
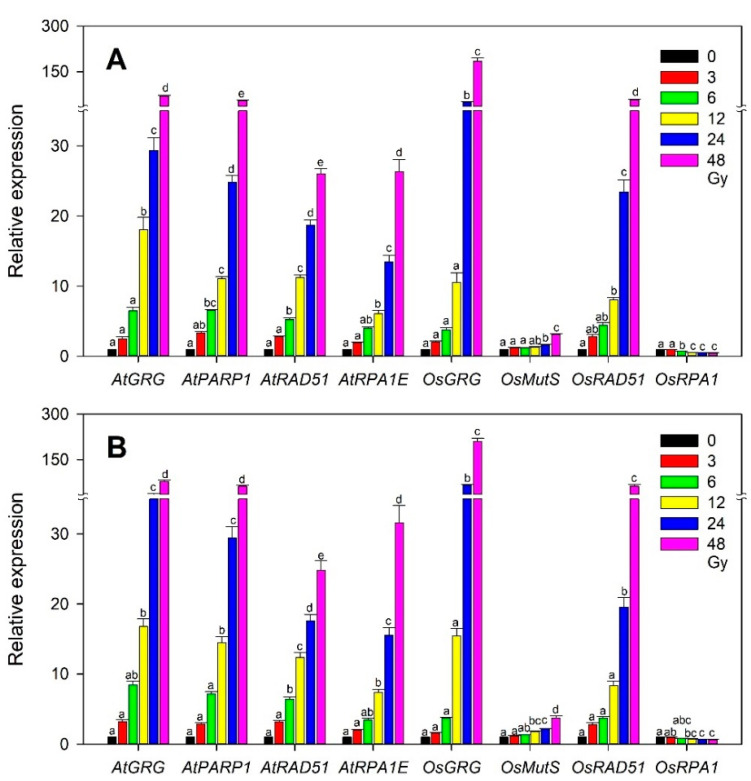
Difference in the dose-dependent transcription of the selected rice and *Arabidopsis* gamma-ray-responsive genes at two developmental stages or seedling ages after sowing. (**A**,**B**) represent 10- and 20-day-old seedlings, respectively. All transcription levels are shown relative to the 0 Gy group. Data are the mean ± SE with *n* = 9 from three independent experiments. Different letters indicate significant differences at *p* < 0.05 (one-way analysis of variance followed by Tukey’s honestly significant difference test).

In *Arabidopsis*, the developmental transition from the vegetative to reproductive stage caused differential physiological changes in plants irradiated with gamma radiation [35], and plants irradiated with an X-ray at different developmental stages showed distinctive expression patterns of DNA repair and epigenetic regulator genes [36]. Even without such a dramatic transition from the vegetative to reproductive stage, a specific developmental stage would differentiate the effects of ionizing radiation at the physiological and transcriptional levels in both monocot and dicot plants. Therefore, we investigated whether different vegetative stages or seedling ages after sowing affect the dose-dependent transcriptions of the selected gamma-ray-inducible marker genes, including *OsGRG*, *OsMutS*, *OsRAD51*, and *OsRPA1*, in rice, or *AtGRG*, *AtPARP1*, *AtRAD51*, and *AtRPA1E* in *Arabidopsis* used for transcriptome-based phytodosimetry models. Although *OsGRG*, *AtGRG*, and *AtPARP1* seemed to be more expressed in the 20-day-old group than in the 10-day-old one, the overall transcription patterns of the genes tested were not substantially different between the two groups in both rice and *Arabidopsis* (Figure 7). This result suggests that the selected marker genes would have little sensitivity to such a developmental difference in the vegetative stage in both plant species, thereby supporting the reliability and statistical confidence of transcriptome-based models. However, transcriptome-based dose–response models need to be further improved by taking into account additional plant species, developmental stages, environmental factors, and genotoxicity-dependent marker genes, or by directly associating transcription with genotoxicity.

## 3. Materials and Methods

### 3.1. Plant Materials and Gamma Irradiation

Rice seeds (*Oryza sativa* L. ssp. *japonica* cvs. Dongjin-byeo and Shindongjin-byeo) were obtained from the National Institute of Agricultural Sciences (Jeollabuk-do, Korea) and surface sterilized for 30 min with 2.5% sodium hypochlorite solution, rinsed three times with sterile deionized water, and incubated at 30 °C for about 2 days. Then, the seeds were subjected to hydroponic cultivation for 12 days under a 16 h light/8 h dark cycle at 23 °C. *Arabidopsis thaliana* seeds of ecotype Columbia-0 were shortly sterilized with 70% ethanol for 1 min and 20% bleach solution for 5 min. They were cultivated on a 1/2 Murashige and Skoog (MS) medium with 1.5% sucrose and 0.65% Phytoagar under the same photocycle and temperature regime. *Arabidopsis* and rice seedlings at three developmental stages (seedling ages after sowing) of 10, 14, and 20 days were irradiated with gamma radiation for 1 h at a dose rate of 3, 6, 12, 24, 48, 100, or 200 Gy h^−1^ using a 3 kCi ^60^Co source at the Advanced Radiation Technology Institute (Jeollabuk-do, Korea). Only aerial parts of the seedlings were pooled immediately after gamma irradiation, frozen with liquid nitrogen, and stored at −80 °C. Unless otherwise noted, the subsequent γH2AX, comet, and RT-qPCR assays were performed using 14-day-old *Arabidopsis* or Shindongjin-byeo seedlings.

### 3.2. γH2AX and Comet Assays

For the γH2AX assays, nuclear proteins were extracted as previously described [4]. To avoid protein degradation and dephosphorylation, a nuclear isolation buffer (0.25 M sucrose, 60 mM KCl, 15 mM NaCl, 5 mM MgCl_2_, 1 mM CaCl_2_, 15 mM 1,4-Piperazinediethanesulfonic acid (PIPES) pH 6.8, 0.8% Triton X-100, and 1 mM Phenylmethanesulfonyl fluoride (PMSF)) was supplemented with a protease inhibitor cocktail (cOmplete ULTRA Tablets; Roche Diagnostics, Mannheim, Germany) and a phosphatase inhibitor (50 mM Na_3_VO_4_ and 30 mM NaF). The protein samples were subjected to 15% sodium dodecyl sulfate-polyacrylamide gel electrophoresis, blotted, and immunodetected with rabbit anti-human γH2AX antibodies (1:1000; Sigma-Aldrich, St. Louis, MO, USA) as previously described [4,24,37]. The band intensities on the immunoblots were determined using ImageJ 1.52v (NIH, Bethesda, MD, USA).

For the comet assays, nuclei were obtained by slicing aerial tissues with a razor blade in 1× phosphate-buffered saline supplemented with 50 mM Ethylenediaminetetraacetic acid (EDTA) on ice [4]. The nuclei were collected by centrifugation and fixed in 1% low melting point agarose on microscope slides precoated with 1% normal melting point agarose. They were then subjected to unwinding in a high alkaline buffer (0.3 M NaOH, 5 mM EDTA pH > 13.0) and electrophoresis in the same solution followed by neutralization in 100 mM Tris-HCl. The slide samples were sequentially washed with 1% Triton X-100 followed by 70% and 96% ethanol, and then stained with propidium iodide solution (2.5 μg mL^−1^). The microscopic images were analyzed using Komet 5.5 image analysis software (Kinetic Imaging, Ltd., Liverpool, UK).

### 3.3. Quantitative Real-Time Polymerase Chain Reaction (RT-qPCR) Analysis

Total RNA was extracted from 10-, 14-, or 20-day-old seedlings using a RNeasy Plant Mini Kit (QIAGEN, Hilden, Germany) and cDNA was synthesized from 1 μg of each RNA sample using oligo(dT) primers and a LaboPass^™^ cDNA Synthesis Kit (Cosmogenetech, Seoul, Korea). The subsequent PCR was performed in a LaboPass^™^ IP pro-*Taq* PCR Mastermix (Cosmogenetech) with 26–30 cycle reactions of 95 °C for 10 s, 58 °C for 10 s, and 72 °C for 1 min using the gene-specific primers (Table 1). In contrast, the quantitative PCR (qPCR) amplifications were performed at 95 °C for 30 s, followed by 40 cycles of 95 °C for 10 s, 58 °C for 10 s, and 72 °C for 1 min with a CFX Connect^™^ Real-Time PCR Detection System (Bio-Rad Laboratories, Hercules, CA, USA) using an iTaq Universal SYBR^®^ Green Supermix (Bio-Rad Laboratories). The amount of cDNA was 1 μL for PCR and 2 μL for qPCR. The relative expression level of each gene was calculated between the control and gamma-irradiated samples using the comparative C_T_ method [38]. The relative mRNA expression data of the three biological replicates in the qPCR were normalized against the reference gene *OsUBi* for rice or *AtACT2* for *Arabidopsis*. *OsUBi*, *OsACT1* and *AtACT2* genes were used as endogenous controls to normalize for differences in the amount of total RNA.

### 3.4. Statistical Analysis

All experiments were repeated three times using the biological replicates harvested after different gamma irradiations. The data were subjected to statistical nonlinear (polynomial) regression analysis and/or one-way analysis of variance followed by Tukey’s honest significance difference test using the statistical and graphical functions of SigmaPlot 12.0 and PASW Statistics 18 (SPSS, Chicago, IL, USA). A *p*-value less than 0.05 was considered significant.

## 4. Conclusions

Currently, transcriptome data are widely used in molecular diagnostics of environmental threats to plants. Species-specific and genome-wide transcriptomic profiles are easily available through RNA-Seq analysis and are applicable to reliable and fast evaluation of stress-induced responses in plants. In this context, our study shows that transcriptome-based phytodosimetry models could be reliable in both monocot and dicot plants as shown in rice and *Arabidopsis*. However, the distinctive genotoxicity- or transcriptome-based models between rice and *Arabidopsis* demonstrated that phytodosimetry of genotoxicity would depend on the relative toxicity specific to a species or an individual. Therefore, since transcriptome-based models are reliable only with their representative materials, additional models using regional representative plant species will help to improve environmental risk assessment of ionizing radiation. In contrast, the effective integration of multiple transcriptome-based models from various plant species needs to be further studied to develop a standardized model of phytodosimetry that is applicable to a broad range of environmental regions.

## Figures and Tables

**Table 1:** Primer sequences used for RT-qPCR. Parentheses represent Rice the Annotation Project Database (RAPdb) or Arabidopsis Genome Initiative (AGI) numbers. *OsUbi* and *OsACT1* in rice, and *AtACT2* in *Arabidopsis* were used as endogenous controls. *OsGRG* and *AtGRG* represent for *Oryza sativa Gamma-ray Responsive Gene* and *Arabidopsis thaliana Gamma-ray Responsive Gene*, respectively.

Gene	Primer Sequence (Forward/Reverse)
*OsACT1* (*Os03g0718100*)	5′-CCTCTTCCAGCCTTCCTTCAT-3′/5′-ACGGCGATAACAGCTCCTCTT-3′
*OsBRCA2* (*Os01g0164900*)	5′-GCAAAATGAAGTAGCTAAGAAG-3′/5′-GTCTGTGCGGTTGCTAAAGG-3′
*OsGRG* (*Os04g0403400*)	5′-CTACTGAAGCCAGAGCCGTTTC-3′/5′-CTAACGATGTCGCAGGCCTATC-3′
*OsH2A* (*Os03g0279200*)	5′-GCCGGGAAGTCCCCCAAGAAG-3′/5′-GACACAAGCACAGATCACAAGG-3′
*OsMutS* (*Os05g0498300*)	5′-ACTTGGTTGGAAAGGCCAATTC-3′/5′-TTCATTGGCTGACACCTGCTC-3′
*OsRAD51* (*Os12g0497300*)	5′-CTTCAGGATACAGCATGAGTTTGC-3′/5′-GTACACCCCCGCTGAAACAC-3′
*OsRPA1* (*Os03g0214100*)	5′-GTTCTCTCCAAGCCCACGAAC-3′/5′-TTGTACGTCCTCAGGTTGCC-3′
*OsUbi* (*Os01g0328400*)	5′-ACCACTTCGACCGCCACTACT-3′/5′-ACGCCTAAGCCTGCTGGTT-3′
*OsWEE1* (*Os02g0135300*)	5′-CCATCTGCGAAAGAAGTCCTG-3′/5′-TTGGGGAGTTTCTCTTGGTG-3′
*AtACT2* (*At3g18780*)	5′-GCCCAGAAGTCTTGTTCCA-3′/5′-CTTGGTGCAAGTGCTGTGAT-3′
*AtGRG* (*At4g22960*)	5′-AGGGTACAAAAGGGCTCACG-3′/5′-TGCGGAACAGGACACAAAGT-3′
*AtRAD51* (*At5g20850*)	5′-TACCGCTCTCTACAGAACAG-3′/5′-ATTCTCTCCTCTGCTCTTCC-3′
*AtRPA1E* (*At4g19130*)	5′-TGGAGAAGTGACGACTGAAGC-3′/5′-ACCTCCAGTTGCGGAACAAT-3′
*AtPARP1* (*At2g31320*)	5′-ACCCATCAGAGGCTCAAACA-3′/5′-ACGCATCTTGATTTGTTCCACA-3′

## Data Availability

Not applicable.
